# Adaptive optics for optical microscopy [Invited]

**DOI:** 10.1364/BOE.479886

**Published:** 2023-03-29

**Authors:** Qinrong Zhang, Qi Hu, Caroline Berlage, Peter Kner, Benjamin Judkewitz, Martin Booth, Na Ji

**Affiliations:** 1 Department of Physics, Department of Molecular & Cellular Biology, University of California, Berkeley, CA 94720, USA; 2 Department of Engineering Science, University of Oxford, Oxford OX1 3PJ, UK; 3 Charité - Universitätsmedizin Berlin, Einstein Center for Neurosciences, NeuroCure Cluster of Excellence, 10117 Berlin, Germany; 4 Humboldt-Universität zu Berlin, Institute for Biology, 10099 Berlin, Germany; 5 School of Electrical and Computer Engineering, University of Georgia, Athens, GA 30602, USA

## Abstract

Optical microscopy is widely used to visualize fine structures. When applied to bioimaging, its performance is often degraded by sample-induced aberrations. In recent years, adaptive optics (AO), originally developed to correct for atmosphere-associated aberrations, has been applied to a wide range of microscopy modalities, enabling high- or super-resolution imaging of biological structure and function in complex tissues. Here, we review classic and recently developed AO techniques and their applications in optical microscopy.

## 1. Introduction

### 1.1 Optical microscopy

Optical microscopy utilizes light to visualize small features beyond the resolving power of our eyes. Light matter interactions can generate image contrast via various mechanisms including reflection, absorption, polarization, and fluorescence [1]. Owing to its noninvasiveness and high spatial resolution, optical microscopy has become an indispensable tool for biomedical research. In particular, recent innovations in fluorescence microscopy [2–4], along with advances in fluorophore design and attachment strategies [5–9], have revolutionized our ability to probe biological structures and dynamics in living organisms.

Based on their illumination and image formation schemes, optical microscopy can be categorized into widefield and point-scanning techniques. In its most basic implementation, a standard widefield fluorescence microscope illuminates samples uniformly, collects the emitted fluorescence with an objective lens, and forms images on a camera. Recording the image of an entire field of view simultaneously, widefield microscopy stands out for its high imaging speed and is often the preferred method for measuring fast dynamics (e.g., membrane voltage dynamics [10]). Furthermore, the simple and low-cost implementation of widefield systems facilitated the development of miniaturized microscopes and their applications in freely moving animals in the past decade [11–13]. Despite these advantages, image contrast is reduced by out-of-focus light in standard widefield microscopy, which limits its applications in complex 3-dimensional (3D) samples. To impart optical sectioning capability to widefield microscopy, one can either physically confine the illumination to the focal plane or computationally remove out-of-focus fluorescence [14]. Physical excitation restriction has been realized by light-sheet microscopy where the illumination is confined to a thin plane [15–19] and total internal reflection fluorescence microscopy where the evanescent illumination field (50 to 200 nm thick) is used for excitation [20]. Alternatively, out-of-focus fluorescence can be removed computationally by using structured illumination to selectively modulate in-focus fluorescence [21–23] or by deconvolution [24].

In point-scanning microscopy, a laser beam is focused and scanned across the sample, and the emitted fluorescence at each position is typically measured by a point detector (e.g., a photomultiplier tube; PMT). An image is then generated by assigning these fluorescence brightness readings to their corresponding spatial locations in the sample. Confocal microscopy focuses the excitation light, typically in the visible spectrum, and detects single-photon excited fluorescence. The out-of-focus fluorescence is blocked by a pinhole that is placed before the detector and optically conjugated to the excitation focus [25,26]. Providing excellent optical sectioning capability, confocal microscopy has lower imaging speed compared to widefield microscopy due to the point-scanning process. Spinning-disk confocal microscopy improves imaging speed by utilizing synchronized scanning of multiple excitation foci and their corresponding confocal pinholes, allowing cameras to be used for detection [27]. Multi-photon microscopes (MPMs) focus near-infrared (NIR) light and generate signal via non-linear optical processes, for example, non-linear absorption and harmonic generation [28–30]. Popular MPM methods include 2-photon fluorescence microscopy (2PFM) [31], 3-photon fluorescence microscopy (3PFM) [32], second or third harmonic generation microscopy [33], and stimulated Raman scattering microscopy [34]. Because the nonlinear signal is only generated within the high-intensity focus, in MPM a confocal pinhole is no longer needed for out-of-focus background rejection, enabling its applications in opaque samples. Moreover, the NIR excitation light also penetrates scattering tissue more effectively than the visible excitation light used in confocal microscopy. Together, these properties make MPMs well suited for deep imaging through scattering tissues [35], with new strategies being advanced to further increasing the imaging speed of MPMs [36].

The spatial resolution of conventional optical microscopy is limited by diffraction to ∼200 nm [37]. Super-resolution microscopy techniques have extended the achievable resolution by as much as an order of magnitude [38]. Three main types of super-resolution microscopy are: single-molecule localization microscopy (SMLM) [39–41], structured illumination microscopy (SIM) [42–45], and stimulated emission depletion (STED) microscopy [46,47]. A widefield microscope, SMLM creates signal sparsity using fluorescent markers that are activated or switched on/off, making spatially inseparable emitters temporally separable for localization with nanometer precision. SIM, also a widefield technique, employs high-frequency structured illumination to down-modulate sample spatial frequencies into the diffraction-limited passband of the microscope. A point-scanning method, STED overlays a diffraction-limited excitation focus with a doughnut-shaped focus that de-excites fluorophores by stimulated emission depletion, producing an effectively sub-diffraction fluorescence excitation volume.

The past decades have witnessed a surge of innovations in optical microscopy. The actual imaging performance metrics, from resolution, speed, sensitivity, contrast, to imaging field of view (FOV) and depth, however, all critically rely on the absence of optical aberrations in the imaging process.

### 1.2 Optical aberration in microscopy

Ideally, a point object forms a point image, so that an image serves as an exact copy of the object. In reality, a microscope images a point object into a 3D volume (i.e., point spread function; PSF) and an image represents the convolution of the PSF and the object [1]. Because of the wave nature of light, the tightest focus and therefore the smallest PSF is formed when all light rays intersect at the same point with the same phase and maximally constructively interfere. This diffraction-limited imaging performance, however, can be difficult to achieve in practice. This is because imperfect optical design, optics, and alignment, as well as optically heterogeneous samples, can all affect the optical field of light in terms of phase, amplitude, and polarization, enlarging PSF and degrading image quality. These perturbations in optical field have to be properly compensated to restore optimal imaging performance [48].

In microscopy, phase deviations are often the most detrimental, because they directly compromise constructive interference in the focusing process and therefore image quality. Amplitude variations come from non-uniform energy attenuation during light propagation and can also result in a degraded focus. Mildly affecting resolution, amplitude variations are often left uncorrected (but see Ref. [49]). Polarization alteration is also neglected in most imaging modalities, but it can be problematic for certain systems that either rely on optimized polarization configurations to function (e.g., SIM and STED) or utilize birefringent optics (e.g., GRIN lenses) [50]. For most imaging setups, it is sufficient to only consider phase distortions of the optical field, more commonly known as wavefront aberrations [51]. A wavefront is a 3D surface formed by points in a wave with the same phase. In an aberration-free imaging system, an ideal diffraction-limited focus is formed by a converging wavefront that is spherical in shape. Deviation away from the spherical form near the focus indicates an aberrated wavefront and prevents the formation of a diffraction-limited focus.

As all microscopy modalities reviewed above involve light focusing (e.g., focusing of fluorescence onto the camera of a widefield microscope, focusing of excitation light in a point-scanning microscope), wavefront aberrations affect all forms of optical microscopy. In general, high numerical aperture (NA) microscopes experience larger aberrations [51], since they experience more higher-order aberrations and further degrade image quality. Super-resolution microscopy, typically using high-NA objectives and aiming to resolve sub-diffractive features, is even more susceptible to aberrations than conventional microscopy [52]. Even with a perfectly designed and aligned microscope, the sample itself, especially biological samples, can introduce optical aberrations due to their inhomogeneous refractive index distributions not matching what the microscope optics (especially the objective) are designed for [53,54].

The effects of aberration on an optical microscope depend on the specifics of its image forming process and light paths. A microscope typically has at least two light paths: one for illumination and the other for detection. Aberrations can be corrected by adaptive optics (AO) employing wavefront shaping technologies (Section 1.3). However, aberration correction is not necessarily needed for all light paths to ensure optimal imaging performance. Most widefield microscopes only require aberration compensation for the detection path because aberrations in the low-NA illumination do not affect image quality. This holds for the super-resolution SMLM, which is built upon a standard widefield microscope. Aberrations do affect the illumination patterns in SIM. The resultant phase and orientation shifts [52,55], however, can be measured and computationally corrected during image reconstruction to avoid artifacts [55,56]. Therefore, the SIM illumination path usually does not need active aberration correction. Light sheet microscopies, especially those designed for subcellular resolution imaging, require correction of aberrations in the illumination path to achieve the desired illumination and maintain slicing selectivity [57,58]. In MPM, the imaging resolution solely relies on the quality of the excitation focus. Therefore, aberration correction is only needed for illumination. Notably, the impact of aberrations and the effect of AO on the signal in MPM increase exponentially with the order of nonlinearity [59]. For confocal microscopy, both excitation and detection paths need to be corrected, to ensure diffraction-limited excitation and pinhole-confined detection of the in-focus signal. STED is a confocal microscope with an additional depletion beam. To achieve desired super resolution, it is key to have optimal contrast of the depletion focus, making aberration correction in the depletion beam essential.

When image-forming light covers a broad wavelength range, one also needs to consider the chromatic aberration of the system. Chromatic aberrations are caused by dispersive materials, whose refractive indices are wavelength dependent and lead to chromatic variations in the image-forming properties of a microscope. Nowadays, chromatically corrected optical elements are easily available for microscopy applications. In addition, biological constituents are usually weakly dispersive in the wavelength range of optical microscopy. As a result, for microscopy imaging of biological samples, it is often sufficient to only correct monochromatic aberrations.

In opaque samples, in addition to aberrations, light scattering [60] further degrades image quality. Light scattering reduces the number of usable photons for excitation or detection, leading to a decreased signal-to-noise/background ratio and limited imaging depth. Both aberration and scattering can be compensated with wavefront shaping technologies. The key difference is that aberration correction restores a diffraction-limited focus formed by un-scattered (i.e., ballistic) photons, whereas scattering control redirects scattered photons in the desired direction. Scattering control and compensation have been reviewed extensively [61–66], and in this article we will focus on how aberrations are corrected.

### 1.3 Adaptive optics

**Fig. 1.**
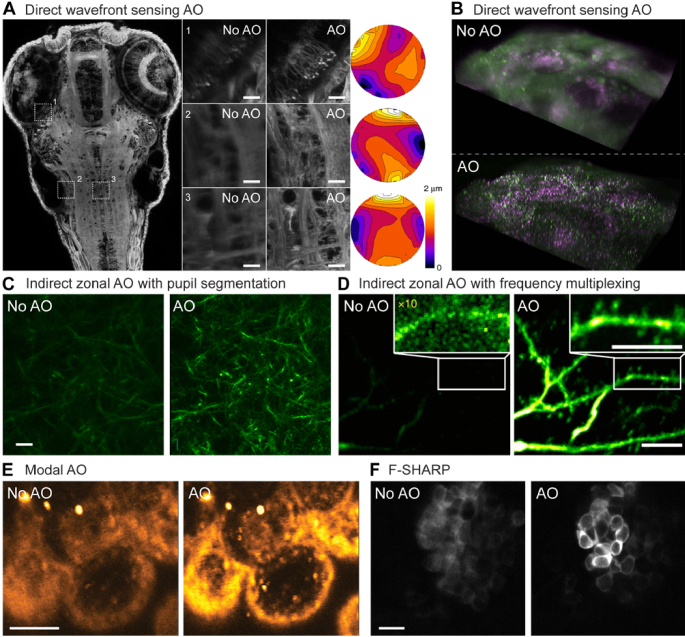
AO improves microscopic images. (A) 2PFM imaging of a living zebrafish larval brain. Left: XY maximum intensity projections after direct wavefront sensing based AO correction. Middle: zoomed-in views before (left) and after (right) AO correction. Right: corresponding corrective wavefronts. Scale bars: 10 µm. (B) Lattice light sheet imaging of endocytosis in a human stem cell-derived organoid before (top) and after (bottom) direct wavefront sensing based AO correction and deconvolution. Green: dynamin; magenta: gene-edited clathrin. (C) 2PFM imaging of mouse brain dendritic processes *in vivo* before (left) and after (right) pupil segmentation based indirect zonal AO. Scale bar: 10 µm. (D) 3PFM imaging of cortical dendritic structures *in vivo* before (left) and after (right) frequency-multiplexing-based indirect zonal AO. Scale bar: 10 µm. (E) Third harmonic generation imaging of a live mouse embryo before (left) and after (right) modal AO. (F) 2PFM imaging of neurons in an 18-day post-fertilization zebrafish brain *in vivo* before (left) and after (right) F-SHARP correction. Panels (A-D,F) reprinted with permission from Refs. [96,58,111,74,135]. Panel E adapted with permission from Ref. [120] © Optica.

AO is a collection of technologies that can actively measure and correct for optical aberrations [48,51,67]. Originally developed for astronomical imaging [68–70], AO has been now widely applied in microscopy (Fig. 1) [51,71] and vision science [72]. There are two key parts in the implementation of AO: aberration determination and aberration correction.

Aberration measurement can be broadly categorized into direct and indirect wavefront sensing methods [48,51,71,73]. Direct wavefront sensing measures aberrations using a wavefront sensor (Section 2). Indirect wavefront sensing characterizes aberrations without a dedicated wavefront sensor. A variety of indirect wavefront sensing approaches have been developed. Wavefront can be measured by segmented zones (zonal methods, Section 3) or as a superposition of modes (modal methods, Section 4). It can also be computationally determined from interferometric focus sensing (Section 5) or by phase retrieval and diversity approaches (Section 6). Recently, machine learning has been used to estimate wavefront aberration (Section 7). In addition, conjugate and multiconjugate AO methods have been developed for correcting spatially-varying aberrations (Section 8). These approaches are discussed in detail in the following sections.

Optical aberrations are compensated by wavefront corrective devices. Once the aberrated wavefront is known, an opposite corrective wavefront can be applied to a corrective device to cancel out the measured aberrations. The most used corrective devices are liquid crystal spatial light modulators (SLM) and deformable mirrors (DM) [48,51]. An SLM contains an array of liquid crystal pixels. By controlling the effective refractive indices of these pixels, the wavefront of light propagating through the liquid crystal (in either transmission or reflection geometries) can be controlled. A DM can have a continuous reflective surface or consist of mirror segments. The advantage of an SLM lies in its large number of pixels, allowing the correction of high-order aberrations. However, SLMs only modulate the wavefront of a specific polarization and therefore are not optimal for wavefront shaping of fluorescence. SLMs also have more limited operating wavelength ranges than DMs. The polarization independence and broadband operation of DMs permit a wider range of applications. In addition, the refresh rate of a DM is generally faster than that of an SLM, and, thus, is preferable for AO methods that require rapid wavefront modulation [74]. There exist other corrective devices including deformable phase plates [75,76], acousto-optic deflectors [77], and digital micromirror devices [78], but they are not as commonly used as SLMs and DMs.

The theory, implementation, and application of AO in astronomy, vision science, and microscopy were comprehensively reviewed in a recent article [48]. With typically static aberrations (or slowly evolving, as in the case of developing embryos) and often high opacity in biological samples, new AO methods have been developed for aberration correction. In the following sections, we review the concept and implementation of these methods, discuss their advantages, their limitations, and their applicability for various microscopy modalities and biological samples.

## 2. Direct wavefront sensing AO and its applications

The most widely used wavefront sensor is the Shack-Hartmann (SH) sensor [79–81]. Based on the principle that a wavefront can be estimated from phase gradient measurements [82], an SH sensor segments the received wavefront into zones and directly measures the local phase gradients of each zone.

**Fig. 2.**
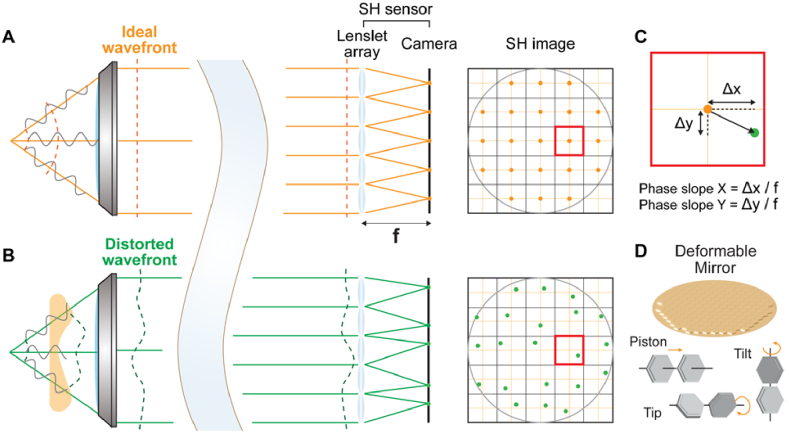
Principle of the Shack-Hartmann (SH) sensor. (A,B) An ideal wavefront (A) and distorted wavefront (B) measured by an SH sensor. (C) 2D local phase gradients calculated from displacements of a segmented focus. (D) Wavefront correction with a deformable mirror with each segment controls piston, tip, and tilt.

An SH sensor is composed of a lenslet array conjugated to the objective back pupil plane and a camera placed at the focal plane of the lenslets. Reaching the SH sensor, a wavefront is segmented and focused by the lenslet array, forming an array of foci (i.e., an SH image) on the camera. For an aberration-free wavefront, the foci are evenly spaced in the SH image (Fig. 2(A)); for a distorted wavefront, the foci are displaced (Fig. 2(B)). Local phase gradients can be calculated from the displacements of the foci centroids from those taken without aberrations (Fig. 2(C)), from which the wavefront can be computationally reconstructed by assuming a continuous wavefront [83,84].

In astronomy, direct wavefront sensing measures aberrations experienced by light from a natural or artificial (e.g., generated by a laser) star. Similarly in microscopy, a light-emitting source or a ‘guide star’ is generated in the sample. While propagating out of the sample, its wavefront accumulates aberrations which are then measured by an SH sensor. Back-scattered or reflected excitation light [85,86], harmonic generation [87], autofluorescence [88], and fluorescence [89] have all been used for direct wavefront sensing. 3D confinement of the guide star is crucial for accurate focal displacement measurements [48]. For this purpose, isolated exogenous fluorescent beads were used as guide stars in widefield microscopy [90,91], confocal microscopy [92], and widefield microendoscopy [93]. Confocal microscopy’s optical sectioning capability enables 3D-confined detection from the guide star, making it possible to employ endogenous signal for wavefront sensing. However, a confocal pinhole filters high-order aberration modes, which may lead to compromised measurement accuracy. It is therefore critical to carefully select the pinhole size for balanced wavefront sensing and optical sectioning [94,95]. In multi-photon microscopy, signal is restricted to a sub-femtoliter focal volume, thus acts as an ideal guide star [89].

Consisting of components with distinct refractive indices (e.g., water, proteins, nuclear acids, lipids) and sometimes possessing curved geometry, biological samples can have complex and spatially varying aberrations. When wavefront complexity exceeds the sampling power of the lenslet array, high-frequency wavefront variations may lead to ambiguous SH foci and inaccurate aberration measurement [96]. The spatial variability may also lead to highly local corrections (i.e., a small isoplanatic patch size). Scanning over a small area instead of using a fixed guide star in the sample and de-scanning the guide star signal before direct wavefront sensing [97], one can accurately measure the averaged wavefront and achieve effective correction over a larger image area [96]. This AO approach (Fig. 1(A),(B)) has been applied with multiple imaging modalities including widefield (optical sectioning SIM) [56], confocal [96,98], lattice light sheet [58,99–102], 2PFM [96,98,103–105], and super-resolution [55,106] microscopy.

The primary advantage of direct wavefront sensing lies in its fast operation. Compared with indirect AO methods that need a sequence of images, direct wavefront sensing can be done in a single camera exposure. This enables direct wavefront sensing (typically at 1kHz [107]) to correct for atmospheric aberrations that rapidly change with turbulence. In microscopy, fast wavefront sensing and correction is also advantageous. Direct wavefront sensing enables high-speed high-resolution imaging of developing embryos [58,96] and over a FOV much larger than the isoplanatic patch size [96]. Its fast operation is also beneficial in reducing photo-toxicity/photo-bleaching. Moreover, because the wavefront is derived from the displacements of the focal array rather than changes in signal brightness or image quality, direct wavefront sensing can be used with time-varying signals (e.g., calcium imaging [56,104]) and is more resistant to sample motion.

The main limitation of direct wavefront sensing is that to acquire a well-defined SH image with a high-quality focal array, the light used for wavefront sensing should be minimally scattered. This becomes challenging when sensing deep in scattering tissues, in which ballistic signal for wavefront sensing is reduced by scattering and overwhelmed by the increased diffuse background. As a result, direct wavefront sensing is usually limited to transparent samples or to the shallow depths of opaque samples. Applying long-wavelength NIR guide stars has extended the application of direct wavefront sensing depths to 700 µm [98] and 800 µm [103] in the mouse brain.

To optimize the sensitivity and ensure the accuracy of direct wavefront sensing, the SH sensor and the corrective device need to be carefully designed and selected, which has been discussed in detail in Ref. [48]. In addition, it is notable that the best achievable performance is limited by system correction, with which the SH reference image is typically acquired. Therefore, non-common-path errors [108] due to the need for a separate wavefront sensing path must be minimized. System correction should compensate for as many optical components as possible in the light path(s) that affect the system imaging quality (discussed in Section 1.2).

## 3. Zonal approach with indirect wavefront sensing and its applications

**Fig. 3.**
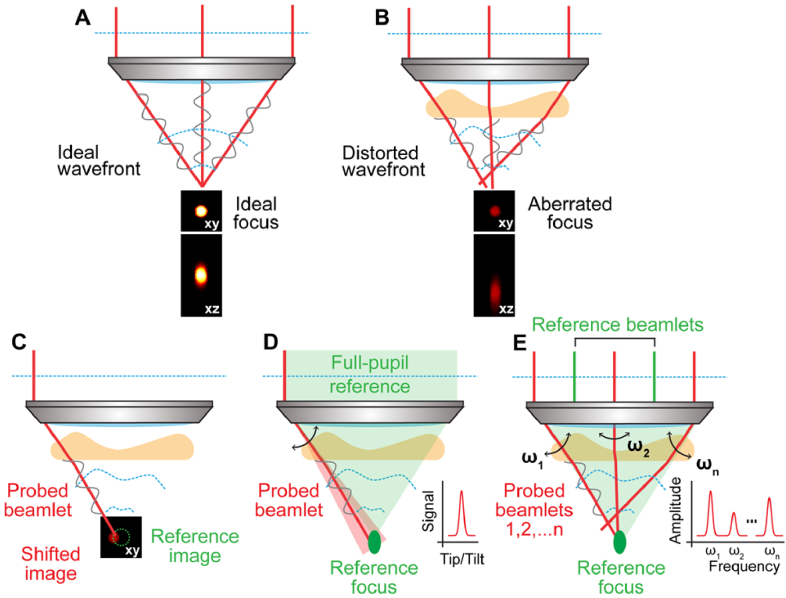
Principle of indirect zonal AO approaches. (A,B) Focus formed from light with an ideal wavefront (A) and a distorted wavefront (B). 2-µm-diameter bead images were normalized to the ideal case. (C) Pupil-segmentation AO method with single-segment illumination. (D) Pupil-segmentation-based AO with full-pupil illumination. (E) Multiplexed aberration measurement.

The local phase gradients of a wavefront can also be indirectly measured based on the focus-forming process in a microscope. A collimated beam of light entering a focusing lens can be considered as a collection of parallel rays. In the absence of aberrations, all light rays are focused to the same point and are all in phase, ensuring maximal constructive interference and generating the tightest and brightest focus (Fig. 3(A)). In the presence of aberrations, the light rays deviate from the ideal focus and/or get phase-shifted so that they no longer maximally overlap or constructively interfere, resulting in an enlarged and dim focus (Fig. 3(B)). Aiming for maximal interference and thus diffraction-limited focusing, a class of indirect wavefront sensing approaches were developed for multiphoton microscopy.

In these approaches, the light beam at the back pupil of the objective is considered as composed of a finite number (<100) of beamlets. Traveling through the aberrating sample, these beamlets and their corresponding wavefront segments pick up phase gradients, which lead to displacements from the diffraction-limited focal position in the focal plane (Fig. 3(B)). Consequently, similar to how an SH sensor measures aberrations, these phase gradients can be calculated from the displacements.

In one implementation for two-photon fluorescence microscopy, individual segments of the back pupil are sequentially illuminated by the excitation light. The beamlet traveling through each segment is scanned to generate a two-photon fluorescence image. For each segment (e.g., corresponding to the “probed beamlet” in Fig. 3(C)), the shift of its image relative to a reference image (e.g., taken by illuminating the entire back pupil) can be used to calculate the phase gradient of its wavefront [109]. With the phase gradients of all segments known, the final wavefront can be algorithmically calculated as in direct wavefront sensing [83]. The relative phase offsets among the wavefront segment can also be experimentally measured by finding offset values that enable maximal constructive interference among the beamlets [109,110]. The measured aberrations are then canceled out employing a corrective device. Correcting either sample-induced or optical system aberrations, this pupil-segmentation AO approach (Fig. 1(C)) allowed diffraction-limited 2PFM imaging at 450 µm depth in the mouse brain *in vivo* [111], enlarged the imaging FOV in 2PFM microendoscopy [112,113], facilitated high-resolution volumetric imaging of the mouse brain *in vivo* with Bessel-focus 2PFM [49], enhanced resolution and signal in scanning laser ophthalmoscopy (confocal) for mouse retinal imaging *in vivo* [114], and improved image quality of widefield microscopy [115].

During the aberration measurement, because the excitation light only illuminates a segment of the pupil (e.g., 1/25 of the back pupil area), the excitation NA is much reduced. As a result, accurate aberration measurement requires samples with axially confined fluorescent features and relatively high focal intensity, preventing it from being applied to samples with dense labeling (can be alleviated by a virtual imaging scheme [116]) or at large depths in scattering tissues. For the latter applications, an alternative approach was developed [117], which follows the same physical picture of focus forming (Fig. 3(D)). Here, the entire pupil is illuminated. The focus can be considered as resulting from the interference between a “probed” beamlet and the rest of the beamlets, with the latter forming a reference focus. Scanning the probed beamlet around the reference by applying phase gradients to the beamlet and recording the fluorescence signal, one finds the gradient values that maximize or minimize fluorescence (for constructive or destructive interference between the probed beamlet and the reference focus, respectively), indicating maximal spatial overlap between the probed beamlet and the reference focus. Repeating the same procedure for all beamlets, one acquires the local phase gradient values across the entire wavefront. The final corrective wavefront can then be determined following the methods described in the previous paragraph. Utilizing the full NA of the microscope objective, this method drastically reduced the power needed for aberration measurement and can be applied to densely labeled samples.

Interfering one beamlet with the rest can lead to small signal variations that require long integration time to measure accurately; serial investigation of all beamlets further prolongs the time needed for aberration measurement. Frequency multiplexing has been used to speed up the process [118]. The phase gradients of half of the beamlets can be probed simultaneously by modulating their phase or intensity at distinct frequencies (ω_1_, ω_2_, …, ω_n_ in Fig. 3(E), typically 100s to 1,000 Hz) while scanning them around the reference focus formed by the rest of the beamlets (Fig. 3(E)). The amount of interference of each modulated beamlet (modulated at ωi) with the reference focus can be directly read from the Fourier transform (FT) of the time-dependent signal trace as the FT magnitude at ω_i_. The gradient values that lead to maximal overlap between the beamlet and the reference focus are determined as ones that maximize the FT magnitude.

Beside the speed gain due to multiplexing, the detection of alternating instead of direct signal offers additional benefit: Because laser-associated noise decreases at higher frequencies, modulating the signal improves the signal to noise ratio. Used with 2PFM, this frequency-multiplexed aberration measurement method recovered diffraction-limited resolution in mouse cortex and zebrafish larvae *in vivo* [118]. Combined with 3PFM, this approach allowed synapse-resolving imaging of cortical and hippocampal neurons and high-resolution imaging of mouse spinal cord neurons *in vivo* at depths (Fig. 1(D)) [74].

With wavefront sensing acquired through sequential measurements, these indirect zonal methods are slower compared to direct wavefront sensing. Because the aberrations of most biological samples do not change rapidly over time [48], ultrafast wavefront sensing is not essential. Moreover, these methods do not require NIR guide stars and can be applied in scattering tissues at depths.

## 4. Modal approach and its applications

**Fig. 4.**
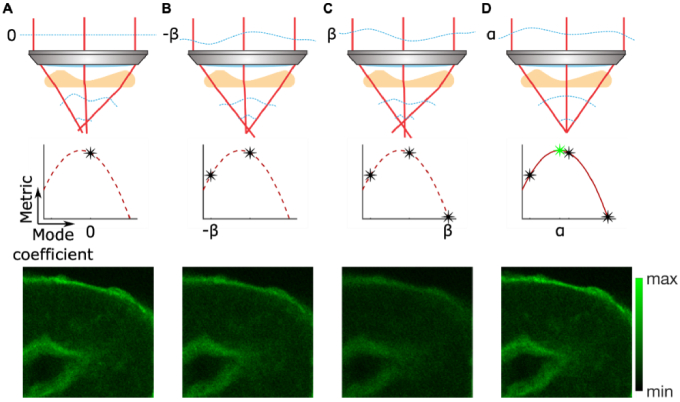
Principle of indirect modal AO approaches. Three images over the same FOV of a mouse kidney section (FluoCells Prepared Slide #3) were captured when (A) no bias, (B) -β, and (C) +β amount of a chosen mode was introduced into the system by the AO corrector. A metric value was calculated for each captured image. (D) A modal based algorithm maximized the metric to compute the optimal correction coefficient α for the mode of interest. The process would then be repeated for different modes.

Indirect zonal methods described above belong to the category of wavefront sensorless AO. Commonly used across microscopy modalities, wavefront sensorless AO methods do not employ a wavefront sensor and therefore are simpler in system design and implementation. A powerful wavefront sensorless method infers the necessary aberration correction indirectly from the properties of a set of aberrated images (Fig. 4). This sensorless AO method is usually implemented through the introduction of aberrations defined in terms of modes and it is hence often referred to as “modal sensorless AO”. The aberrated images are acquired through the application of known aberrations (termed “bias aberrations”) using the adaptive element. These bias aberrations are defined in terms of modes – often Zernike polynomials, although other modes such as deformable mirror deformation modes are also used. Through careful choice of the applied bias aberrations, one can estimate the unknown specimen aberration through a calculation that uses a mathematical model of the image formation process.

The image is a function of both the object structures and the point spread function (PSF) of the microscope. In an incoherent imaging system, such as a fluorescence microscope, the image is given by the three-dimensional convolution of the fluorophore distribution and the PSF. Information about the aberration is contained entirely within the PSF, but this PSF is not in general directly accessible from the images due to the convolution with the specimen structure. The PSF may only be retrieved if the object is point-like (Section 6), such as a small fluorescent bead (but see Section 5). For this reason, it is necessary to acquire a number of images, each with a different bias aberration applied, in order to estimate and correct the aberration independently of the specimen structure.

Modal aberration correction is based on the principle that the image has optimum quality only when the aberrations have been fully corrected. This process therefore requires the definition of an image-derived metric that is proxy for image quality. This metric is minimized or maximized, as appropriate, in order to find the optimum aberration correction. The definition of the optimization metric depends upon the image formation process of the microscope. For example, in confocal or non-linear microscopes (like two-photon or harmonic generation microscopes), the total image intensity (equivalently, the sum of pixel values) is an appropriate metric (Fig. 1(E)) [119–125], as this quantity is decreased with all aberration modes that affect image quality. This metric would not however be appropriate in a widefield microscope, where the total image intensity is invariant with aberration content. In this case, a metric related to spatial frequency content or image sharpness is suitable [126–129]. By constructing a mathematical model of the optimization metric as a function of aberration content, one can derive an efficient method for estimating the correction aberration, in which as few as 2N + 1 images are required to correct N aberration modes. Note that there are many examples of model-free optimization of aberration correction, such as using stochastic search methods, but these tend to be much less efficient in terms of number of measurements required for correction [130–132].

Various modal sensorless methods have been deployed across a wide range of microscopes. The methods offer a number of advantages over alternative AO approaches: (1) The practical implementation is usually simpler, as they require only the addition of an adaptive correction element to the microscope and no other components, such as a wavefront sensor. (2) Modal methods can work with any adaptive element, including continuous deformable mirrors, segmented mirrors and liquid crystal spatial light modulators. (3) As the aberration information is inferred from images, the method is highly versatile and not limited to specific microscopy modalities or choice of specimens. (4) As there is no separate aberration sensing path, the method does not suffer from so-called “non-common path” aberrations. Any aberrations that arise from the optical system or the specimen can be sensed using these methods.

Some relative drawbacks of the modal methods include: (1) The method is ideally suited to the correction of a small number (up to tens) of modes, but would be less effective for more complex aberrations. (2) The range of efficient operation is typically limited by the range of approximations used in the model of the optimization function. While this covers many realistic aberrations encountered in microscopy, it does mean that for larger aberrations, more biased images would have to be acquired for effective operation. (3) Similar to the indirect zonal methods described above, the modal method is usually slower than direct sensing methods. The speed of measurement/correction is determined by the rate at which the microscope can acquire the biased images, as computation is not a limiting factor. However, this speed is not generally a problem as many specimens that are mostly static on the timescales of imaging experiments.

One of the challenges in implementation of efficient modal correction methods has been that it has been necessary to define a different method for each microscope modality, such as the need to choose an appropriate optimization metric for a particular type of microscope. This complication has been somewhat alleviated in recent years, through the move towards a more universal framework for sensorless AO. This has included the introduction of wavelet-based multi-scale metrics that can cope with different imaging properties [133] and a broader range of bias aberration modes has been considered, including hybrid zonal-modal combinations [134]. Furthermore, developments in machine learning based sensorless AO are opening up more advanced possibilities (Section 7).

## 5. Interferometric focus sensing methods

Many of the methods described thus far are well suited for correcting low spatial frequency aberrations, i.e., refractive index variations that vary slowly over space. However, when imaging deeper into tissue, the spatial scale of turbidity decreases while the number of modes required to correct it increases. To measure and correct such turbidity efficiently, it may be helpful to rephrase the problem and take a conceptually different approach: instead of determining the wavefront in the Fourier plane, we can aim to measure it at the focal plane after propagation through the tissue.

This is of course fundamentally equivalent: If we can determine the complex-valued electric field at the focal plane (i.e., the electric field PSF, E_PSF_), we can calculate the correction pattern using a Fourier transform. This ultimately allows us to arbitrarily select the number of measured modes, independent of the wavefront shaping device.

**Fig. 5.**
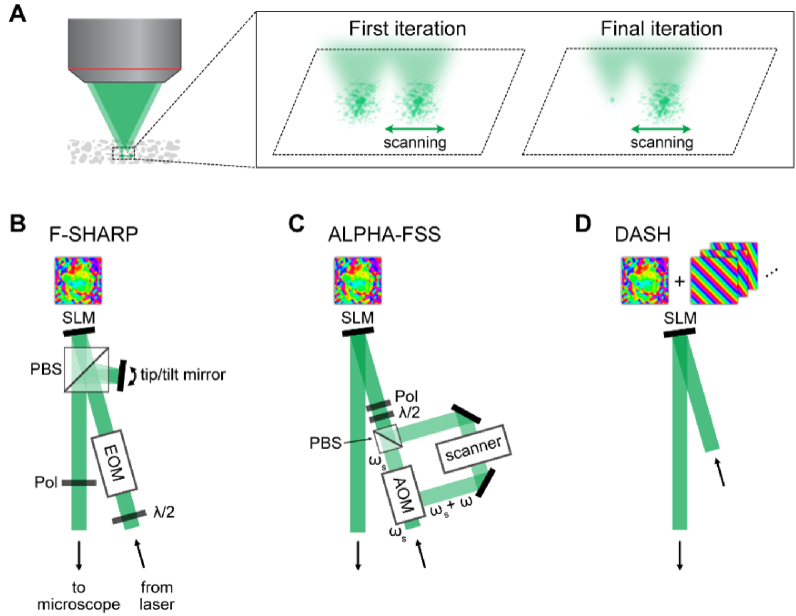
Principle and implementations of interferometric focus sensing. (A) Focus sensing methods use a stationary and a scanning beam to measure the E_PSF_. The calculated correction pattern is used to optimize one of the beams, which iteratively improves the measurement. (B)-(D): Different implementations of interferometric focus sensing. SLM: spatial light modulator, PBS: polarizing beam splitter, Pol: linear polarizer, λ/2: half-wave plate, EOM: electro-optic modulator, AOM: acousto-optic modulator introducing a frequency shift ω, L: lens.

These ‘focus sensing’ methods separate the excitation light into two separate beams and scan one beam against the other. This is analogous to the imaging process in scanning microscopy, where the excitation light probes the sample. In focus sensing, however, it is not the sample that is being probed, but a copy of the excitation beam itself. Using nonlinear excitation and multiple intensity measurements at different phase delays between the two beams, the E_PSF_ can be determined. This measurement is not perfect; In the same way that an aberrated PSF distorts the image of a sample, using an aberrated focus for focus sensing leads to an imperfect measurement. Nevertheless, this measurement can be used to apply a correction pattern on the SLM, which makes the sampling beam gradually more point-like and decreases the error of a subsequent E_PSF_ measurement. Within a few (typically 2-4) iterations, the corrected beam approaches the diffraction limit and the interferometric measurement converges to the E_PSF_. This process is shown in Fig. 5(A).

There are multiple implementations of this process. It was initially introduced as focus scanning holographic aberration probing (F-SHARP, Fig. 1(F)) [135], where the excitation light is separated using a polarizing beam splitter (Fig. 5(B)). One beam is reflected off an SLM and remains stationary, while the other is scanned in two dimensions using a tip/tilt mirror. An electro-optic modulator (EOM) changes the relative phase of the two beams in discrete steps, which yields phase information from intensity measurements (for details, see [135]). These components can be used as a modular add-on to a standard MPM.

A similar approach, termed ALPHA-FSS (analog lock-in phase detection for focus sensing and shaping) [136], uses analog phase modulation and lock-in detection with the help of an acousto-optic modulator instead of discrete phase stepping (Fig. 5(C)).

DASH (dynamic adaptive scattering compensation holography) is a comparable technique developed by May et al. [137]. Instead of splitting the beam spatially, they superimpose a grating pattern for scanning with a stationary correction pattern on the SLM, shown in Fig. 5(D). Phase stepping is also performed by the SLM. The DASH algorithm updates the SLM pattern not only after a full E_PSF_ measurement, but after every mode. This approach, together with a simplified setup, requires fewer iterations, but the scanning rate is limited by the relatively slow update speed of the SLM.

All of these methods determine the correction pattern for a single location in the sample. Due to the intrinsic short-range correlations within the tissue (the so-called optical memory effect [138–140]), it also remains a good approximation for a surrounding region, also known as the isoplanatic patch. The size of this region, however, typically decreases for increasing numbers of corrected modes [141]. One solution is to conjugate the correcting device to the dominant scattering layer, for example the thinned mouse skull [142–144]. This can increase the corrected FOV by nearly an order of magnitude. Alternatively, it is possible to determine correction patterns in multiple positions and, using a large SLM in a sample conjugate configuration, display them simultaneously [145].

When used in combination with 3P excitation, F-SHARP and ALPHA-FSS [136,146] profit from the increased penetration depth of longer wavelength light and reduced background of the higher order nonlinear excitation. Additionally, it leads to two advantages: First, unlike 2P approaches, 3P focus sensing methods are able to determine the aberrations even for a homogeneously fluorescent, three-dimensional sample with strong contributions from outside the focal plane. Second, theory predicts that for optical non-linearities (e.g. multiphoton absorption) of order n, the estimated PSF is taken to the 2(n-1)th power in each iteration. For 2P F-SHARP, this results in an improvement to the third power, while 3P F-SHARP improves to the fifth power, requiring fewer iterations [146].

Focus sensing methods have been used to image microglia in mouse hippocampal tissue slices up to a depth of 530 µm [137,145] and cortical neurons through a craniotomy [135,146] as well as the thinned skull [144]. Recently, the combination of 3P excitation and ALPHA-FSS with conjugate AO and remote focusing has enabled an effective correction over large volumes of cortical neurons below the intact skull up to a depth of 750 µm, as well as high resolution imaging of hippocampal neurons through a craniotomy up to 1.1 mm deep [136].

At such large depths, in addition to aberration correction, interferometric focus sensing methods also correct for scattering. Other methods have been developed for scattering control and compensation, as reviewed elsewhere [61–66]. These are, for example, based on different wavefront modulation schemes [61,147] or time-gated complex-field maps of backscattered waves [148,149]. Interferometric focus sensing enables the fast measurement of large numbers of modes without the need for additional sensors or guide stars. Additionally, they can be employed even for strongly aberrated wavefronts. For this reason, they can lead to higher signal enhancements in scattering tissue when compared to other AO methods. At the same time, the high number of corrected modes leads to a small corrected FOV, which remains one of the main limitations. Nevertheless, the aberration measurement is fast and takes only a few seconds at each position. In the future, this could be used to acquire multiple corrections and update the correction pattern during scanning with fast segmented deformable mirrors.

## 6. Adaptive optics using phase retrieval and phase diversity approaches

While interferometric focus sensing methods directly measure the phase of a wavefront, there are other strategies that can derive the phase information from intensity measurements. One such method involves using the Fourier-Transform relationship between the coherent Pupil function and the coherent PSF in a microscope to determine the wavefront through measurements of the PSF [150]. Because cameras record the intensity, the phase information – the wavefront – is lost. Phase retrieval provides a computational method for reconstructing the phase of the pupil from the recorded intensity PSF [151].

In phase retrieval algorithms, a solution for the phase is sought that is consistent with the constraints in both the Fourier (pupil) plane and the image plane. Typically, these constraints are the extent of the pupil in the Fourier plane and the image intensity in the image plane. The phase retrieval problem was first solved using the Gerchberg-Saxton algorithm. Other solution approaches have been developed including other alternating projection (projection onto convex sets) methods, gradient-descent algorithms, and convex relaxation algorithms [152–155]. Phase retrieval results can be improved by acquiring multiple images which correspond to the same pupil or overlapping pupils. For example, the object can be defocused or the illumination angle in a coherent imaging system can be shifted to shift the pupil.

In fluorescence microscopy, the wavefront in the back pupil plane can be retrieved by imaging a subdiffraction fluorescent object. This was first demonstrated by Hanser et al. who measured the wavefront in the back pupil plane for a widefield fluorescence microscope [156,157]. They used subdiffraction fluorescent beads to measure the three-dimensional PSF then calculated the phase retrieved wavefront from a subset of the 3D image stack. The use of out-of-focus slices of the PSF not only provides multiple images, averaging noise in the images, it also reduces the error introduced by artifacts which do not change with focus (i.e. dust on the camera window) and provides additional information on the aberrations by spreading out the PSF over more pixels effectively providing higher signal-to-noise for the higher frequency features of the PSF. Hanser et al. use a scalar model of the PSF which will affect the accuracy for high NA systems. This work has been extended to a vectorial PSF model which reduces the residual wavefront error by a factor of 2 to 3 [158]. Compared to measurements of the wavefront by a Shack-Hartmann wavefront sensor or inferred through sensorless AO, phase retrieval can provide a much more detailed measurement of the wavefront. A typical wavefront measured with a Shack-Hartmann wavefront sensor will contain a few hundred pixels [159]. Modal sensorless AO might measure the first 20 Zernike modes. Phase retrieval measures the wavefront with the same number of pixels used in the field of view. So even a moderately sized image will produce a wavefront with thousands of pixels.

**Fig. 6.**
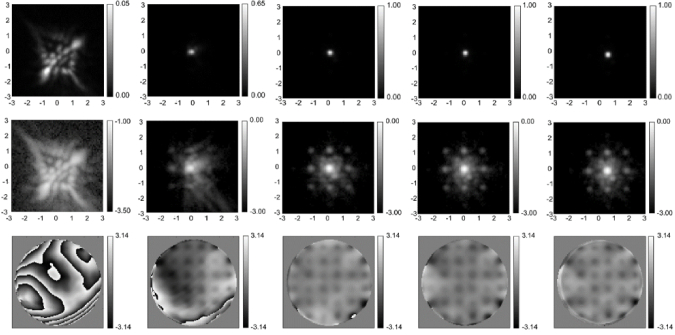
Iterative correction of the microscope point spread function using phase retrieval and AO. Top row: In focus image of the PSF, linear scale. Second row: same as first row but on a log scale. Third row: phase in the back pupil plane calculated from phase retrieval with the tip, tilt and focus terms removed. First column: before correction; DM actuators all set to zero volts. Each successive column is after a round of iteration. The final Strehl ratio is 0.78. From Ref. [160].

The phase-retrieved wavefront can then be used to correct the wavefront in an AO system [160,161]. In Ref. [157], the Gerchberg-Saxton algorithm was used to calculate the wavefront from a 3D PSF measured from -2 to +2 microns relative to focus. Phase retrieval was performed on a 256 × 256 pixel image and the resulting wavefront has more than 10,000 pixels. Starting with a wavefront with several waves of aberration, an increase of more than 10× in the peak intensity of the PSF and correction to a Strehl ratio of 0.78 was demonstrated (Fig. 6). However, the Gerchberg-Saxton algorithm is slow and not guaranteed to converge to the optimal solution. AO correction with different phase retrieval algorithms is investigated in Ref. [161]. They achieved Strehl ratios greater than 0.9 although the best algorithm, a convex relaxation algorithm using the Extended Nijboer-Zernike formulation of the PSF is computationally more intensive than FFT based alternating projection algorithms such as the Gerchberg-Saxton algorithm.

Phase retrieval has been used in SMLM to improve the accuracy of the PSF localization [162,163]. In this approach, single molecule blinking events are assigned an axial position in a biplane imaging system based on a PSF model. An averaged 3D PSF is then generated from the single molecule data which is used to create a pupil wavefront through phase retrieval. Then this wavefront generates a more accurate reference PSF. The process is iterated until the 3D PSF model no longer changes. The model is then used to localize the blinking events in 3D through a cross correlation with the 3D PSF. The localization using the phase retrieved PSF is then more accurate than localization using an ideal PSF or a PSF generated from fluorescent bead data. The wavefront from these phase-retrieved PSFs could then be used with a deformable mirror to improve the SMLM PSF as has been shown using sensorless AO methods [164,165]. This phase retrieval method has been extended to 4Pi SMLM microscopy [166]. In this case the wavefronts for the opposing pupils are generated from independent measurements and then used to construct the 4Pi PSF.

Because phase retrieval assumes a Fourier Transform relationship between the image plane and pupil plane, a coherent source is required for phase retrieval. In a fluorescence microscope, a subdiffraction fluorescent object is used as the coherent source. Another approach, phase diversity, can be used to calculate the wavefront and an estimate of the object from extended objects in incoherent imaging systems [167].

In Phase Diversity, two or more images are acquired with known aberrations applied to the wavefront. The object and wavefront are then estimated through the minimization of a cost function that compares the measured images to calculated images based on the wavefront and object. For the case of Gaussian noise, the object can be eliminated from the calculation of the cost function [168]. The case of phase diversity for 3D imaging in microscopy has been considered in [169]. Phase diversity is closely related to blind deconvolution approaches in which both the object and the aberrations are calculated from a series of images. Various blind deconvolution algorithms have been used in microscopy although typically instead of, rather than with, adaptive optics [170,171]. In one early approach to correcting aberrations with deconvolution, the aberrations were calculated from a refractive index map derived from DIC images [172]. The wavefront was then calculated by ray tracing through the refractive index map, and the PSF was calculated from the wavefront for use in deconvolution.

A useful application of phase retrieval and phase diversity is to measure the DM influence functions [173–175]. In a typical AO application with a wavefront sensor, the wavefront sensor is used to characterize the DM. In many microscopy AO systems, sensorless AO is used and a wavefront sensor is not available to measure the influence functions. In these cases, phase retrieval or phase diversity can be used to characterize the DM. In [173], the influence functions were measured by performing phase retrieval on a 3D PSF measured from a fluorescent bead as in [156,157]. In [175], a mirror was placed in the focal plane of the objective and a brightfield reflectance PSF was measured. A few planes relative to the focal plane were measured by moving the mirror. In [174], multiframe blind deconvolution was used to determine the influence functions from 2D images of a fluorescent bead. A fluorescence bead was measured with all actuators sequentially actuated, and the point object and the wavefronts from all actuators were then determined.

## 7. Machine-learning and computational approaches

Designing faster, more efficient and versatile AO methods is an ongoing quest. Many computational approaches have been developed to assist in the implementation of aberration compensation.

Computational AO methods have been developed mainly for coherent optical systems. With access to complex-valued images in such systems, aberrations can be computed and compensated during image reconstruction [176,177] or by adaptive optics hardware [178]. The advantages of such methods are that fewer sample exposures are required and the sampling of the pupil aberration is not limited by the AO actuators but only by the detection camera. However, due to the reliance of such methods on access to complex-valued images, their applications are less versatile and not universally applicable to all forms of microscopy.

Machine learning (ML) has been vastly developed in the 21st century driven by big data and increasing compute capabilities. Among its various approaches, supervised learning (SL) is widely used as it is easily implemented and has proved to be flexible and effective. SL algorithms are designed to learn the statistical relationships between inputs and outputs [179] and are useful especially when the relationships are difficult to be expressed explicitly in mathematical terms.

SL algorithms are normally in the form of neural networks (NN) where the inputs and outputs are connected by layers of neurons containing trainable parameters (such as weights and biases) and non-linear activation functions. Multiple pairs of input-output datasets are collected experimentally or generated synthetically in order to train networks. During training, the network generates an output for each input; the differences between the network generated outputs and the ground truth (labels) are used to update the trainable parameters in the NN. After multiple iterations (epochs) of training, the trainable parameters converge to steady states and the network is optimized such that it computes outputs similar to the ground truth. Such a properly trained network models the relationship between inputs and outputs and can be used to predict outputs if inputs are provided. In an ideal scenario, the training dataset should encompass all the possible data cases for which the NN is likely to be applied, in order to ensure broad applicability. Though the training process can be relatively time-consuming, once a network is fully trained, computing an output from an input using the network can be very straightforward and fast.

One common application of SL algorithms is to compute the solution of the inverse problem of a well-defined forward problem. Pupil phase aberration retrieval for an imaging system is one such problem. There is a good mathematical forward model to generate a PSF from a given pupil field; however, the inverse process – accurately estimating the pupil phase from the PSF – is more difficult. This challenge has motivated many developments using SL algorithms to design phase retrieval and sensorless AO methods.

One difficulty in the implementation of such a SL sensorless AO method on a microscope is to obtain sufficient training data that encompasses all possible cases that may be encountered in practice. Due to physical limitations such as the lifespan of biological samples and the time and effort required to collect data from a microscope, it is unlikely that one can obtain all the training data in real systems. Many researchers have therefore limited their investigations to a simple case, using 3D PSFs (or multiple 2D sections through these PSFs) as the inputs to determine the pupil phase aberrations, and tested their NN based method through simulations only [180–182] or experiments [183–185]. By restricting the data to PSFs, the scope of analysis and thus the required training dataset is much reduced compared to using arbitrary specimens. Some approaches used training data collected experimentally [183,184] while Ref. [185] showed that a network can be trained on simulated data and tested in real imaging systems. To obtain 3D PSFs, the experimental set-ups of such approaches required multiple photo-detectors to simultaneously obtain multiple layers of the 3D PSF stack [184] or introduced different amount of defocus such that multiple 2D PSFs at different layers were collected sequentially [183,185].

Although shown to be effective, such methods working solely on PSFs were not versatile since they either required special optical designs or point like objects. To design a ML algorithm working on images other than PSFs, Ref. [186] proposed a reinforcement learning (RL) based algorithm and demonstrated it on fiber-like tissue phantoms. However, the method still involved iterative correction and was not shown to be more efficient or effective than many other non-ML driven sensorless AO algorithms. References [187] and [188] used a different approach by incorporating physical understandings of light propagations and imaging processes into the method design. Both cases involved untrained neural networks to generate sample structures and thus determine the wavefront phase. However, such methods were reported to require a few minutes of network convergence, limiting their potential in live imaging applications.

Images can be mathematically expressed as a function of the sample structure and PSFs. In many common microscopes, such as fluorescence microscopes, the imaging process is incoherent and can be represented by the convolution between a specimen function and the PSF. Using physical understanding of the imaging process and the Fourier convolutional theorem, PSF-related information can be readily separated from pairs of images by deconvolution-like processes. Both Refs. [189,190] used this concept to design SL algorithms for retrieving phase from non point-like objects. While Ref. [189] limited their discussion to simulations in non-microscope imaging systems, Ref. [190] demonstrated the method on a range of experimental microscopes for live biomedical imaging and showed its better efficiency and advantages compared to non-ML sensorless AO methods.

In addition to these concepts using ML algorithms for phase retrieval and subsequent aberration correction, NNs are frequently applied to other image processing applications such as denoising and deconvolution. These processes could be readily combined with AO in future developments. With fast advancements in ML and ongoing increases in computational capabilities, more NN-based AO methods with improved performance and versatility are expected to be developed for use in more microscopes in the future.

## 8. Additional considerations

In most implementations of AO, the wavefront correction element is located in a plane conjugate to the back pupil plane (“pupil AO”). This allows for perfect correction at a single point or for an extended field of view with spatially-invariant aberrations. However, sample-induced aberrations in general vary across the field of view because the light from different points in the field travels through different parts of the sample. In scanning microscopy techniques, the aberrations can, in principle, be corrected separately for each point or area-by-area in the field of view, although this approach can be time-consuming. Alternatively, a multi-pupil AO approach separated the pupil images for different sample regions on the corrective SLM with a prism, corrected them separately, and then recombine with another prism [191]. In widefield microscopy techniques, the whole field of view is imaged at once. If the aberrations are corrected for a point in the field of view, only the isoplanatic patch around that point will be well-corrected. To image a larger area, an average correction can be applied. In principle, the multi-pupil AO approach can also be applied to widefield microscopy, although additional considerations/complexities are necessitated by the fluorescence light’s bandwidth and mixture of polarization states.

To fully correct field dependent aberrations, ideally, the correction should be applied in a plane conjugate to the aberrations. For a thick biological sample that aberrates the wavefront throughout light propagation, this would require that the wavefront correction element be three-dimensional with a controllable phase for each voxel. This concept has been modelled [192] and was demonstrated using optical phase conjugation in a Lithium Niobate crystal [193].

A more practical approach to correcting field-dependent aberrations is to place a wavefront correction element conjugate to the layer that is most aberrating – conjugate AO – or to place multiple correction elements conjugate to the different layers between the focal plane and the objective – multiconjugate AO. Conjugate AO has been implemented with different imaging modalities. In widefield microscopy, high-resolution imaging of a USAF target and mammal elastic cartilage was achieved with a partitioned aperture wavefront (PAW) sensor and a DM conjugate to a phase screen above the sample [194]. In 2PFM, through-skull imaging of the mouse brain was demonstrated using a DM conjugate to the intact skull [143]. To maintain the conjugation between correction and the layer to be corrected, remote focusing has been incorporated to achieve large-volume wavefront shaping and demonstrate improved imaging through the mouse skull [136,195]. Multiconjugate AO has been modelled for microscopy modalities to determine the possible performance gain over pupil AO [192,196,197]. One approach [192,197] uses the 3D refractive index map to calculate the maximum correction achievable with a given number of correctors. Another method [196] determines the corrections that maximize the average Strehl ratio across the field of view. Multiconjugate AO has been demonstrated in both ophthalmology [198,199] and microscopy [200,201], with the microscopy systems taking advantage of newly available deformable phase plates [75,76], which simplify the optical setup. One challenge with conjugate and multiconjugate AO is placing the DM(s) in appropriate planes for the maximal benefit. In the case of multiconjugate AO, effective control of multiple correction devices is an additional problem that requires the development of new strategies for wavefront measurement and correction. It is worth noting that in practice, (multi)conjugate AO methods work best for samples with clearly defined dominant aberrating layer(s), a condition that does not apply to most biological samples.

Yet another location to place the wavefront correction device is a plane that is conjugated to the focal plane of the objective (“focal AO”), as demonstrated in microscopy modalities such as AO lattice light sheet microscopy and 2PFM with a Bessel focus [49,58]. In both cases, the excitation light has a small footprint at the pupil plane. Phase correction in the focal plane corrects for both amplitude and phase distortions in the pupil plane, thus has superior performance to pupil AO [49].

Finally, the ultimate goal of implementing AO in optical microscopy is to remove aberration and restore the ideal PSF of the imaging system. Whereas resolution is invariably improved by AO, the associated changes in signal are more complex. For structures within the aberration-free focal plane, their signals usually increase (but see below). The improved axial resolution would cause the signal of structures outside the aberration-free focal plane to decrease. Together, these effects enable AO to improve image contrast. For specific microscopy modalities, the signal of in-focus structures may decrease after AO due to its specific generation mechanism. For STED, an increase in resolution can be accompanied by the reduction in fluorescence signal due to a better confined focus [127]. For third harmonic generation (THG) microscopy, minimal aberrations may not necessarily maximize the signal when several interfaces are near the focus due to the coherent nature of THG [120].

## 9. Conclusion and outlook

Capable of non-invasive imaging with submicron spatial resolution, optical microscopy allows biological investigations under physiological conditions. Biological systems, however, are heterogeneous mixtures of components that produce optical aberrations, compromising imaging signal, resolution, and contrast. AO can correct these aberrations and is therefore essential for high-fidelity microscopic investigations in living organisms. This article reviews AO techniques developed for optical microscopy, including direct wavefront sensing, indirect zonal wavefront measurement, indirect modal wavefront sensing, interferometric focus sensing, phase retrieval and diversity, and machine learning based approaches. Combined with a variety of optical microscopy modalities, these techniques have achieved great successes in restoring the ideal imaging performance in complex samples.

Going beyond demonstration-of-principle experiments, we expect broader applications of AO to biology-centered inquiries and new insights that it will bring to key questions in the biomedical fields. To make this happen, we need hardware-based AO modules with lower cost, more compact design, better software integration, and more robust performance, as well as computation-based AO tools that are plug-and-play. Furthermore, it is often the lack of practical experiences rather than technical barriers that prevents a wider adoption of AO. We therefore encourage the AO microscopy community to provide detailed guidelines for routine applications of AO in the future.

## Data Availability

No data were generated or analyzed in the presented research.
